# Supplementary material for: Coordination Chemistry of Phosphate Groups in Systems Including Copper(II) Ions, Phosphoethanolamine and Pyrimidine Nucleotides
Source: Int J Mol Sci. 2022 Nov 8;23(22):13718. doi: 10.3390/ijms232213718 (PMC9691210; doi:10.3390/ijms232213718)
Supplement: Supplementary file 1 [file ijms-23-13718-s001.zip › ijms-1991862-supplementary.pdf]

Supplementary materials

Coordination chemistry of phosphate groups in systems including copper(II) ions, phosphoethanolamine and pyrimidine nucleotides

Malwina Gabryel-Skrodzka, Martyna Nowak, Anna Teubert and Renata Jastrzab

The set of EPR, UV-Vis, ^{13}C NMR, ^{31}P NMR spectra of the MLH_4L` type complexes as well as corresponding to the free ligand ^{13}C NMR and ^{31}P NMR spectra

1. **Cu(enP)H_4(TMP) pH=2.5**
2. **Cu(enP)H_4(UMP) pH=3.0**
3. **Cu(enP)H_4(CMP) pH=2.5**

Cu(enP)H_4(TMP) pH=2.5

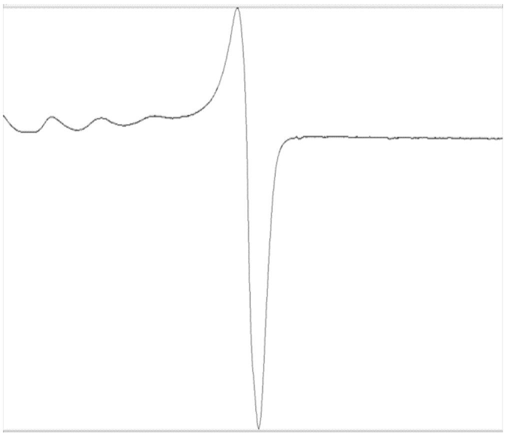

Figure S1. EPR spectrum

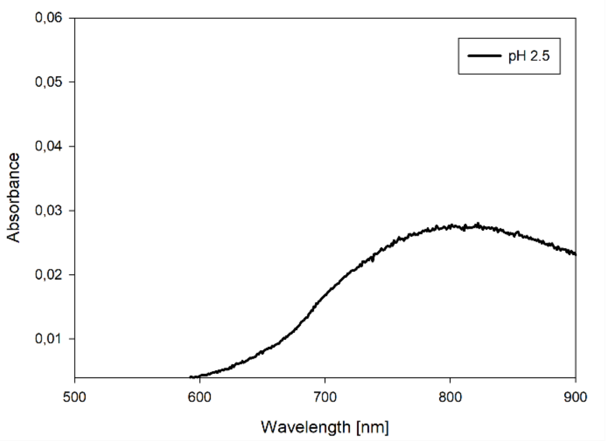

Figure S2. UV-Vis spectrum (C=0.002 M)

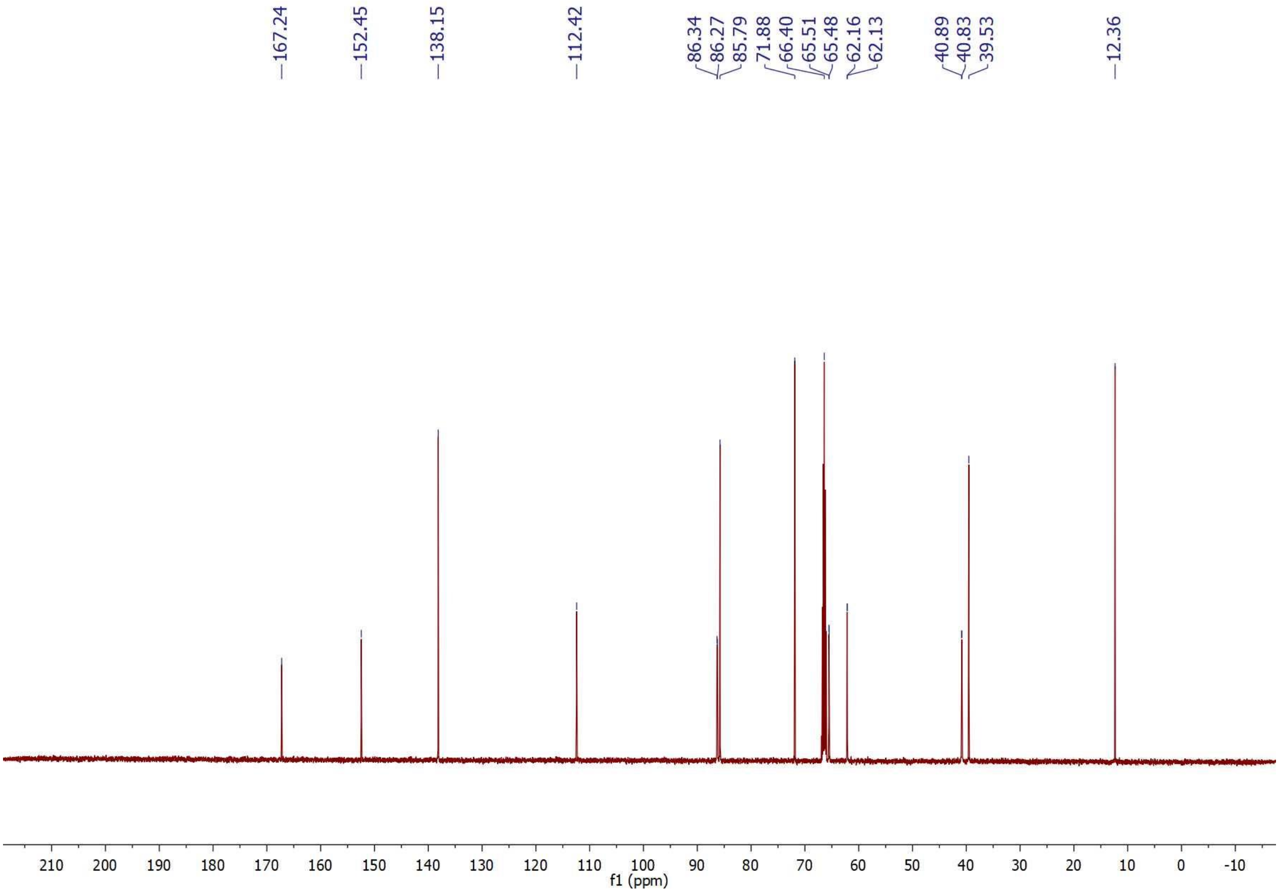

Figure S3. Cu(enP)H_4(TMP) ^{13}C NMR spectrum

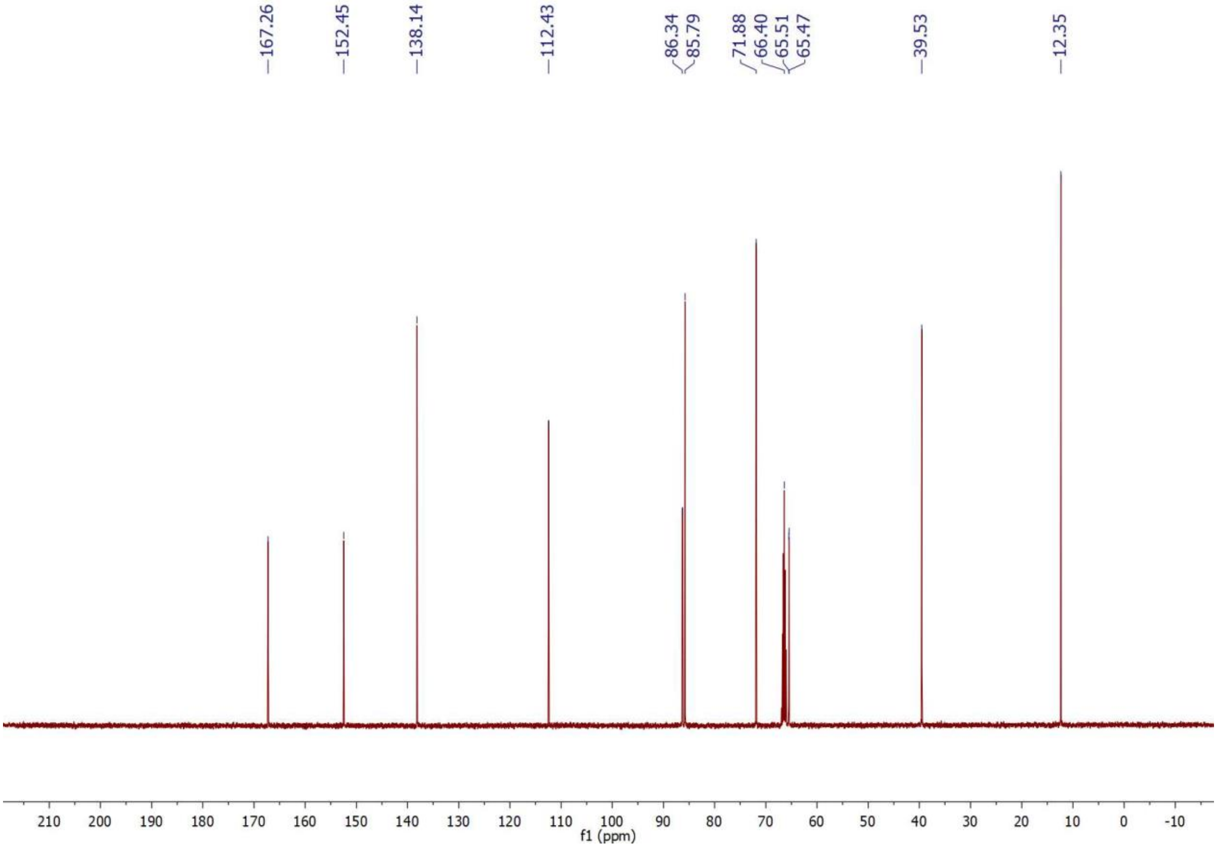

Figure S4. TMP ^{13}C NMR spectrum

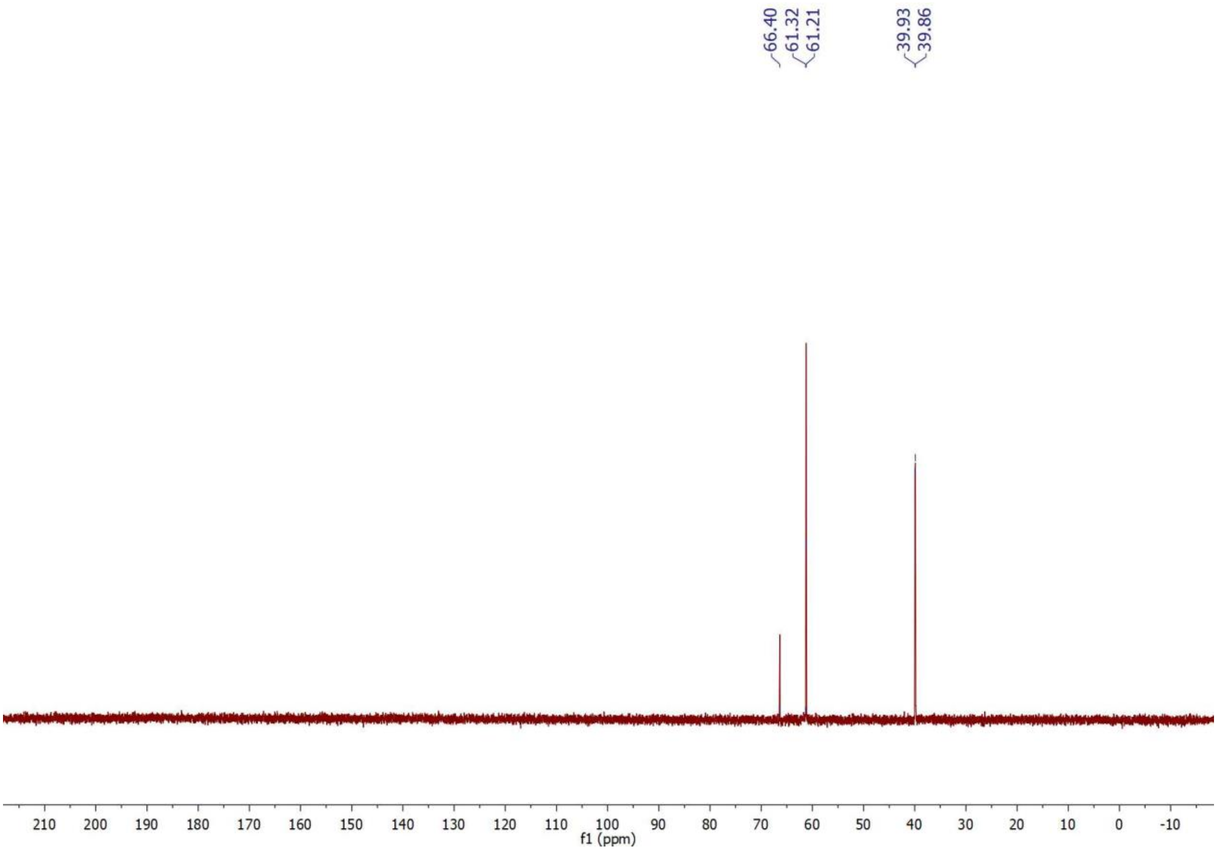

Figure S5. enP ^{13}C NMR spectrum

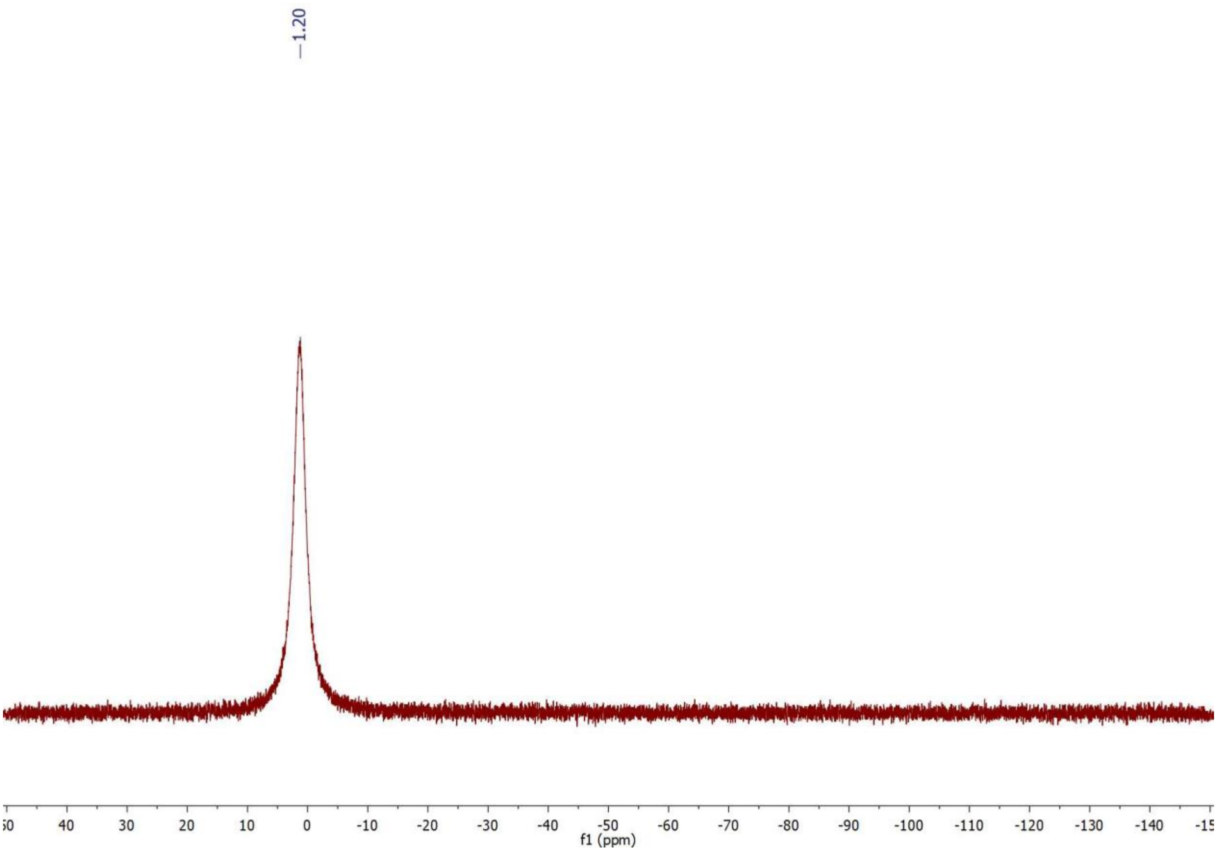

Figure S6. Cu(enP)H_4(TMP) ^{31} P NMR spectrum

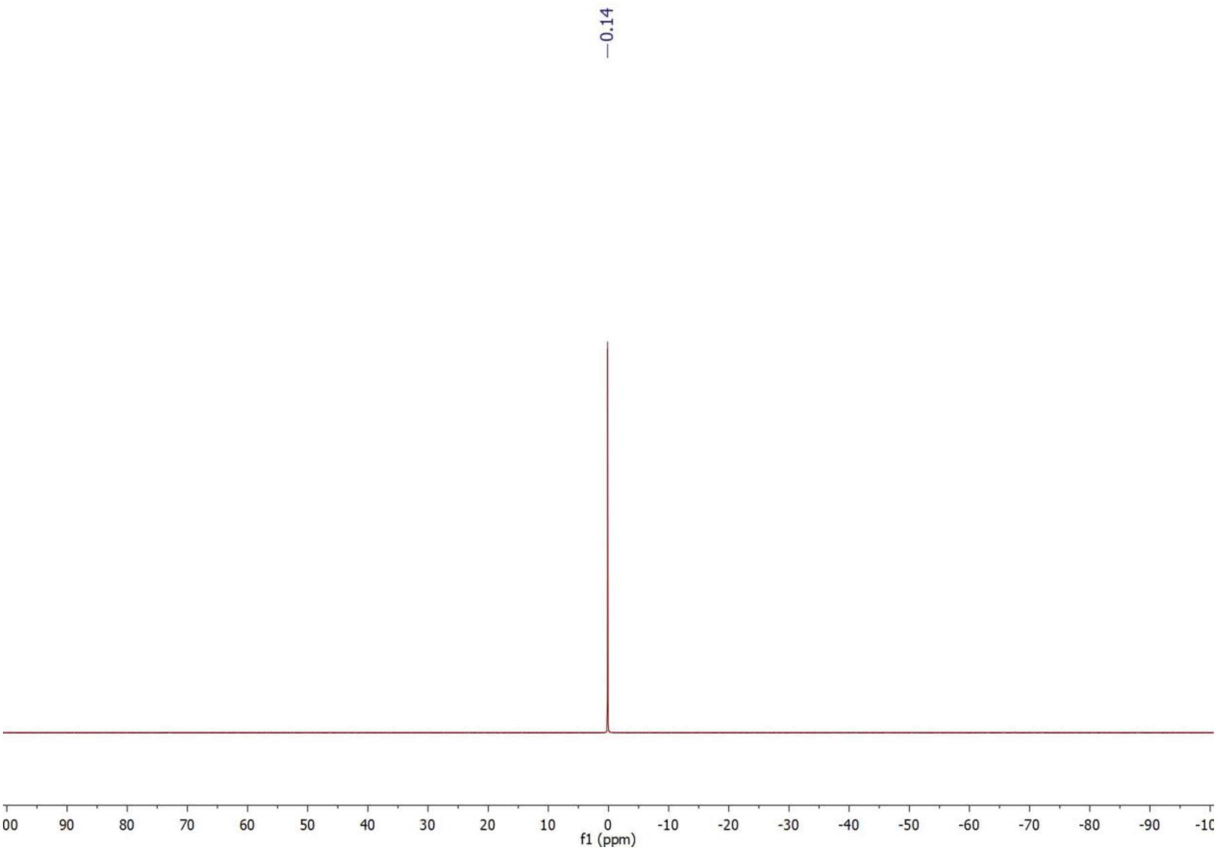

Figure S7. TMP ^{31} P NMR spectrum

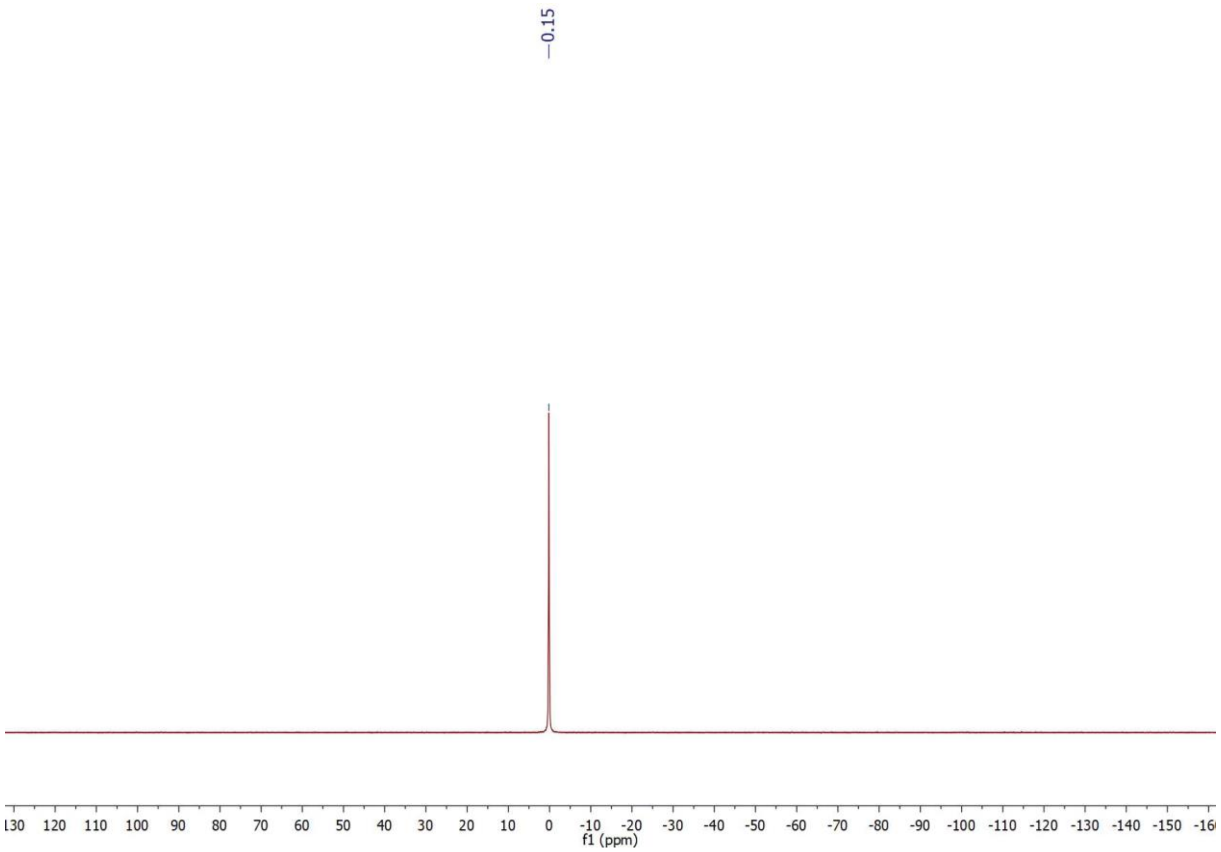

Figure S8. enP ^{31} P NMR spectrum

Cu(enP)H_4(UMP) pH=3.0

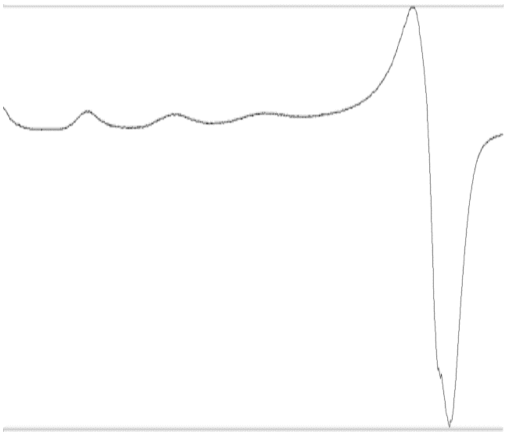

Figure S9. EPR spectrum

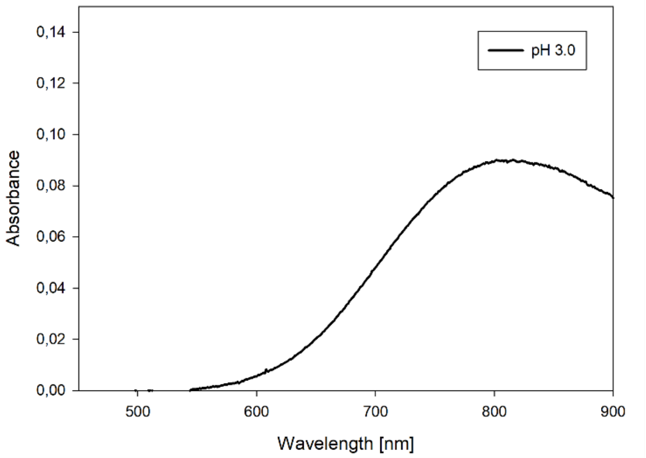

Figure S10. UV-Vis spectrum (C=0.01 M)

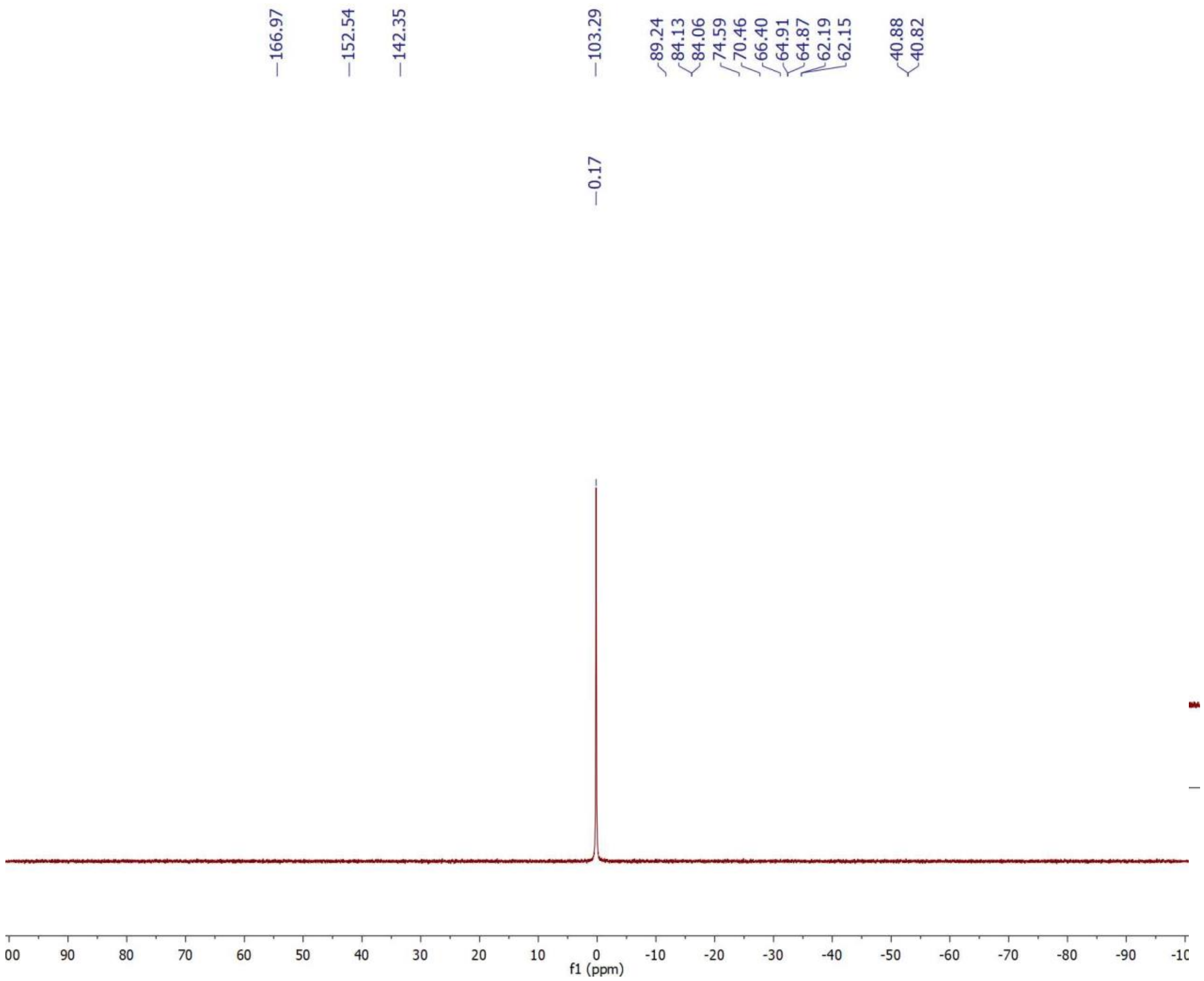

Figure S11. Cu(enP)H_4(UMP) ^{13} C NMR spectrum

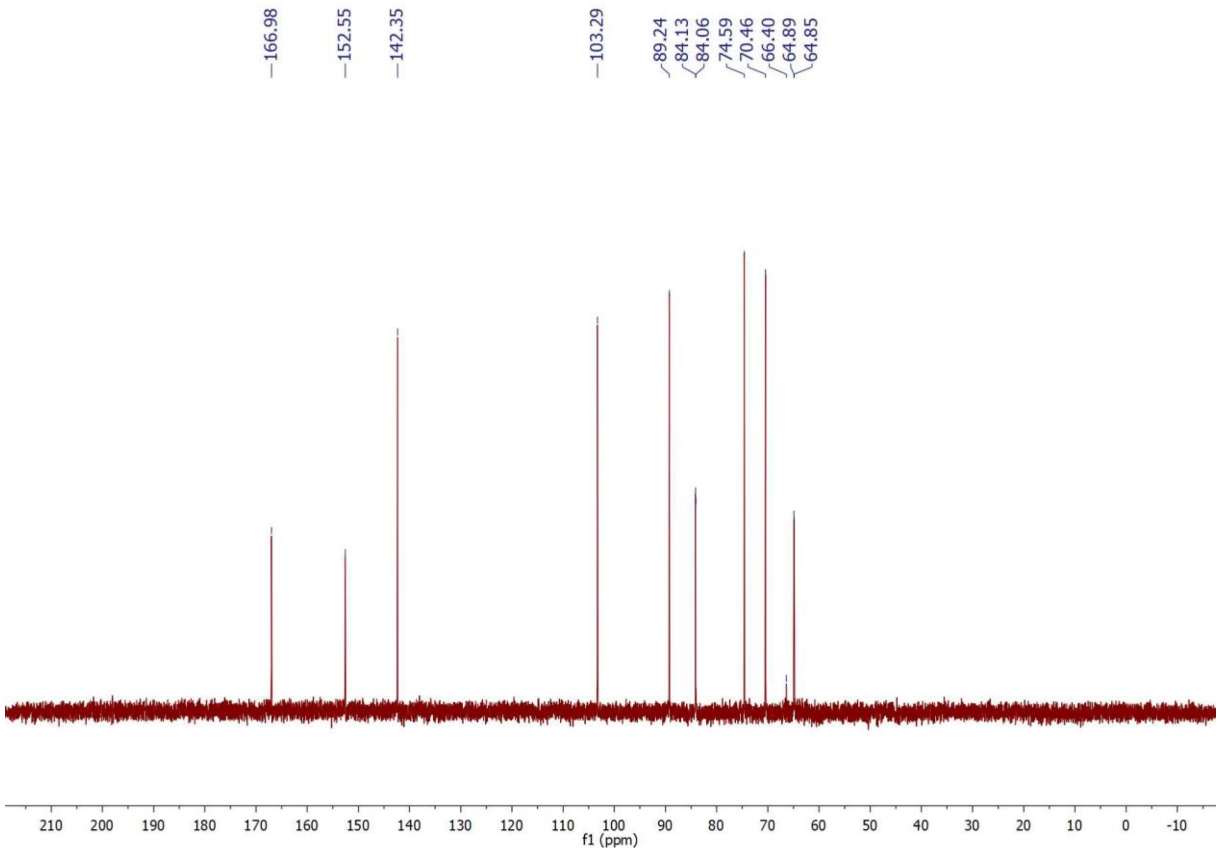

Figure S12. UMP ^{13}C NMR spectrum

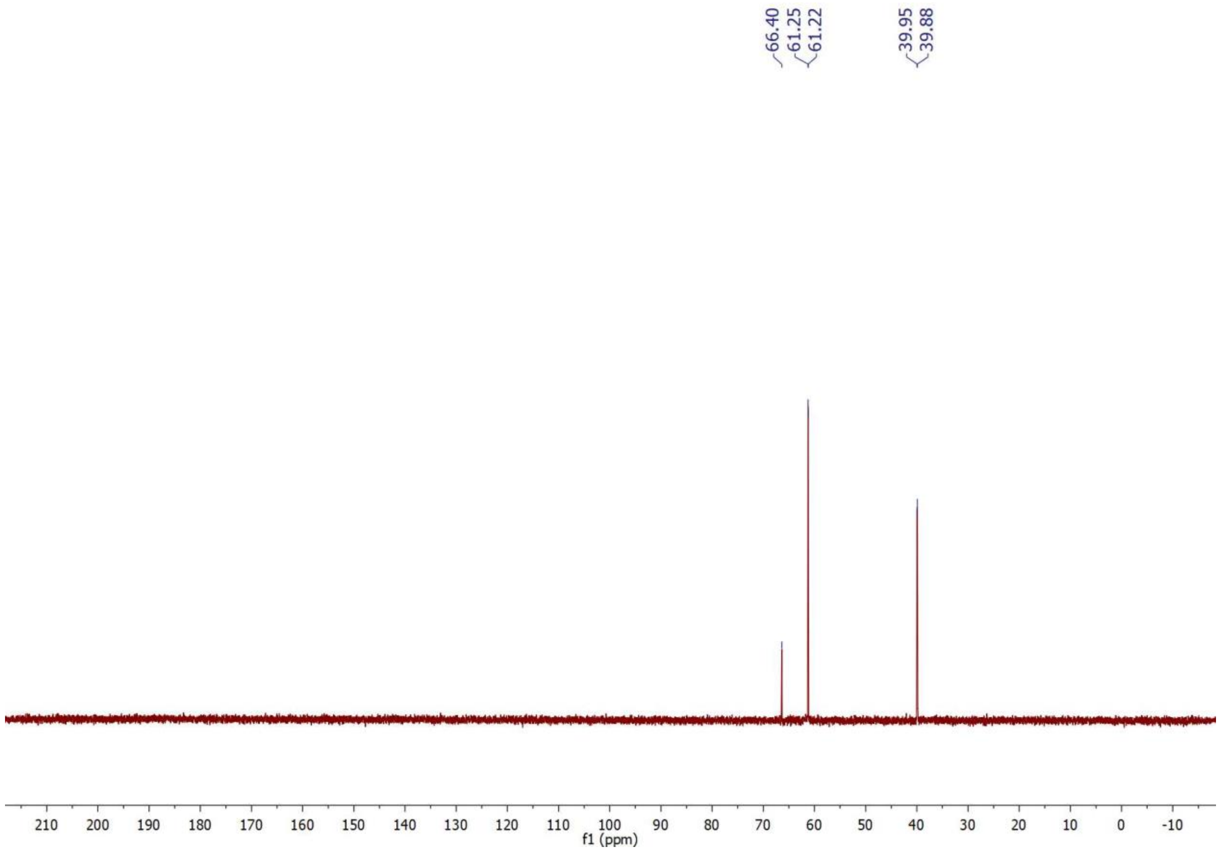

Figure S13. enP ^{13}C NMR spectrum

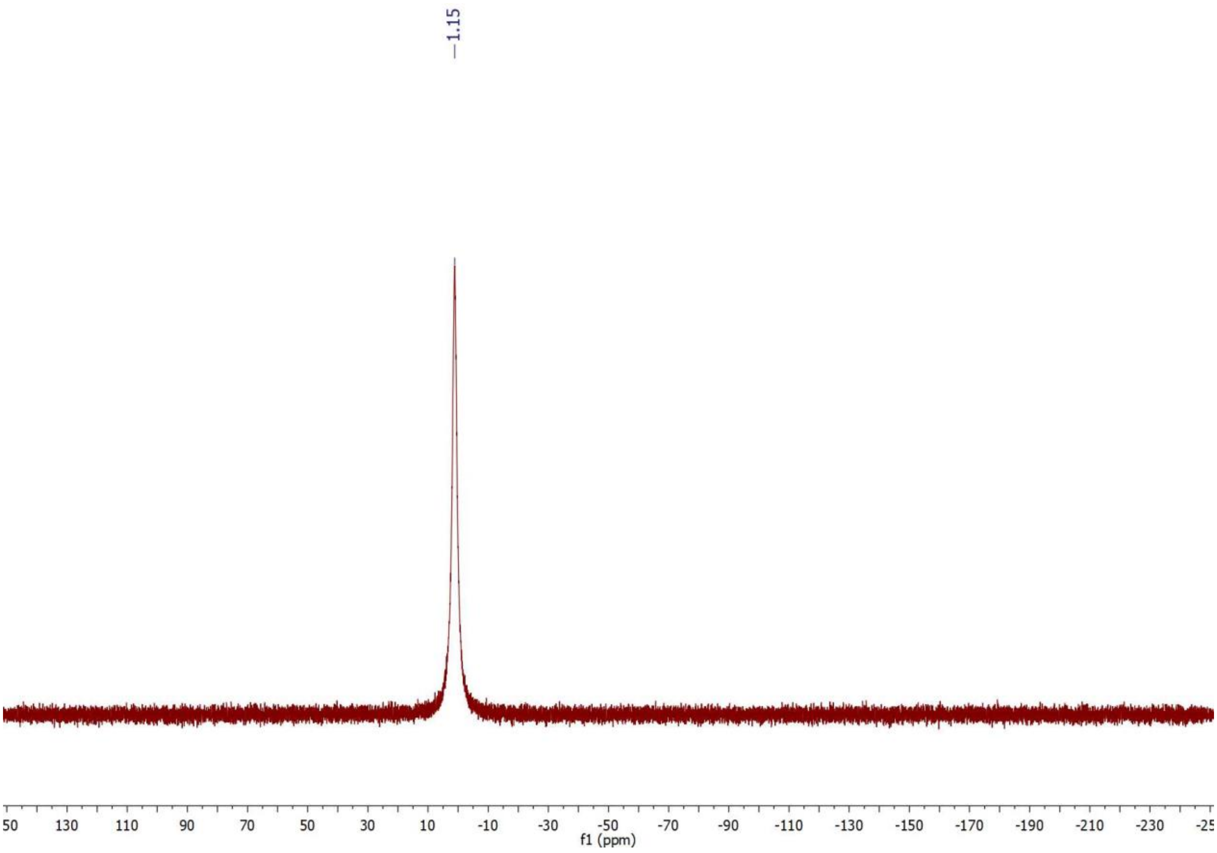

Figure S14. Cu(enP)H_4(UMP) ^{31} P NMR spectrum

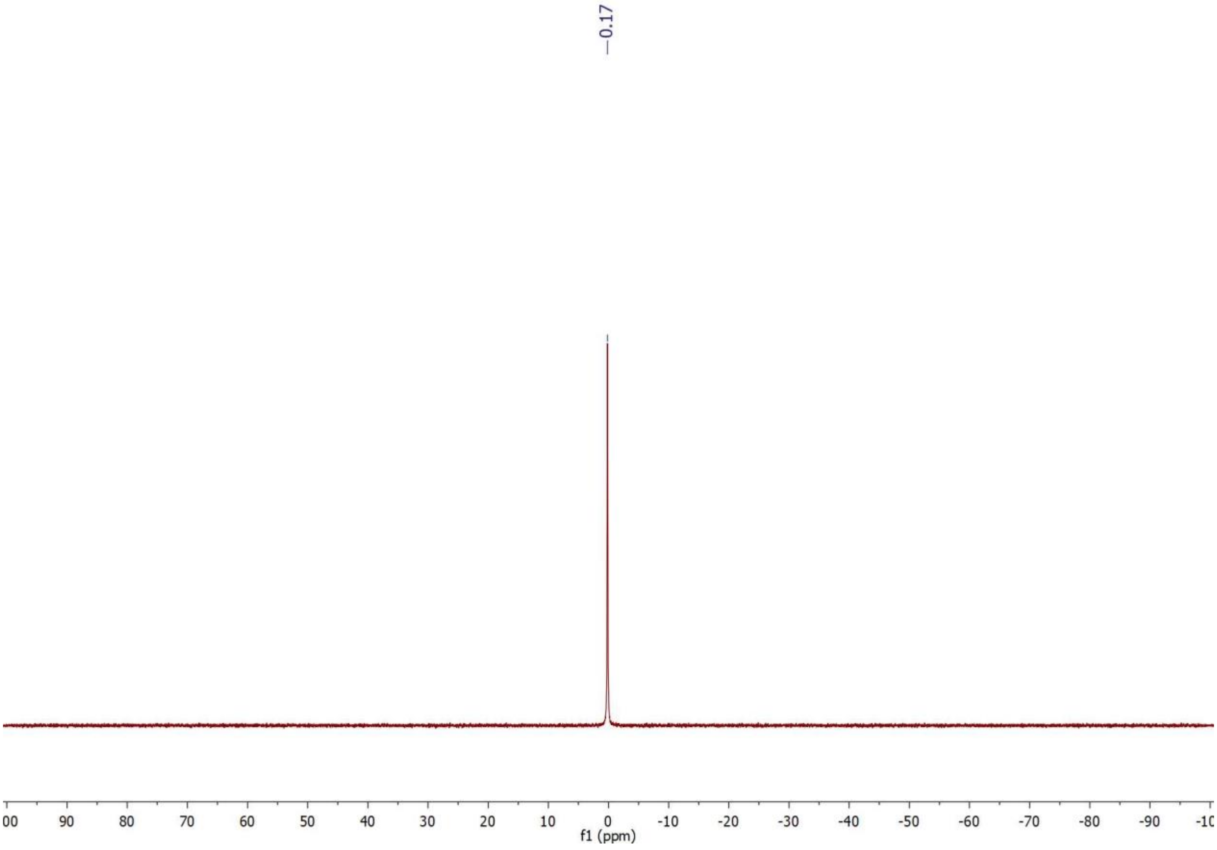

Figure S15. UMP ^{31} P NMR spectrum

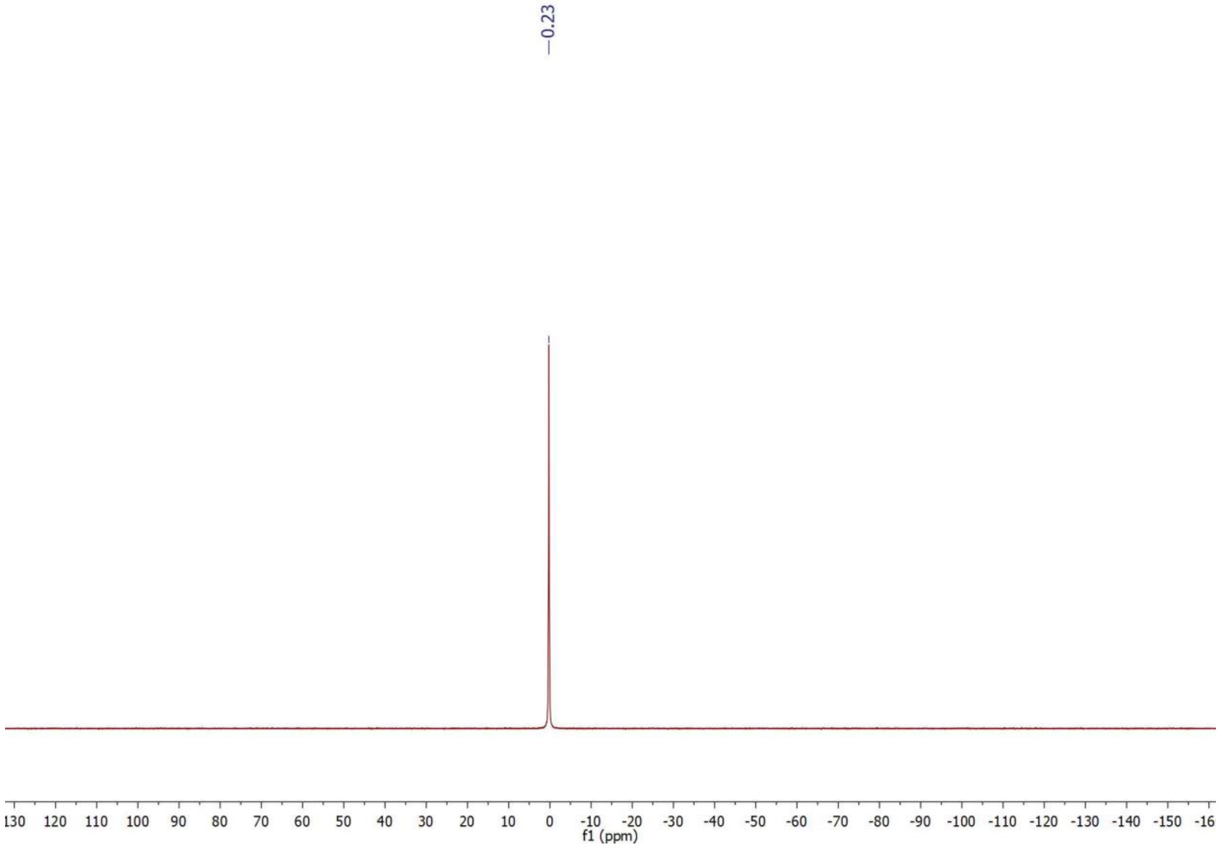

Figure S16. enP [31] P NMR spectrum

Cu(enP)H_4(CMP) pH = 2.5

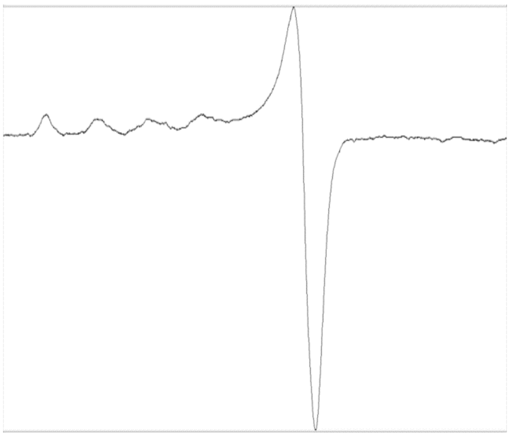

Figure S17. EPR spectrum

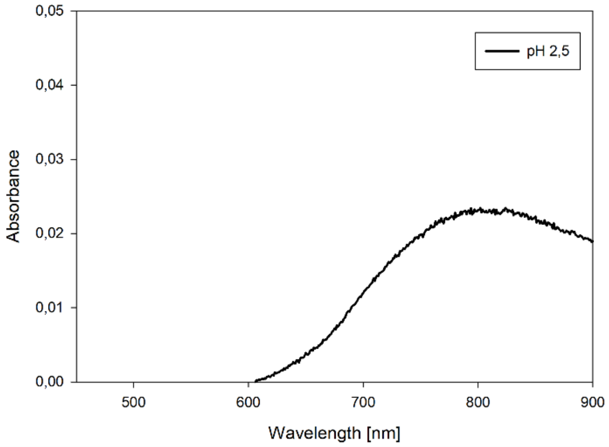

Figure S18. UV-Vis spectrum (C=0.002 M)

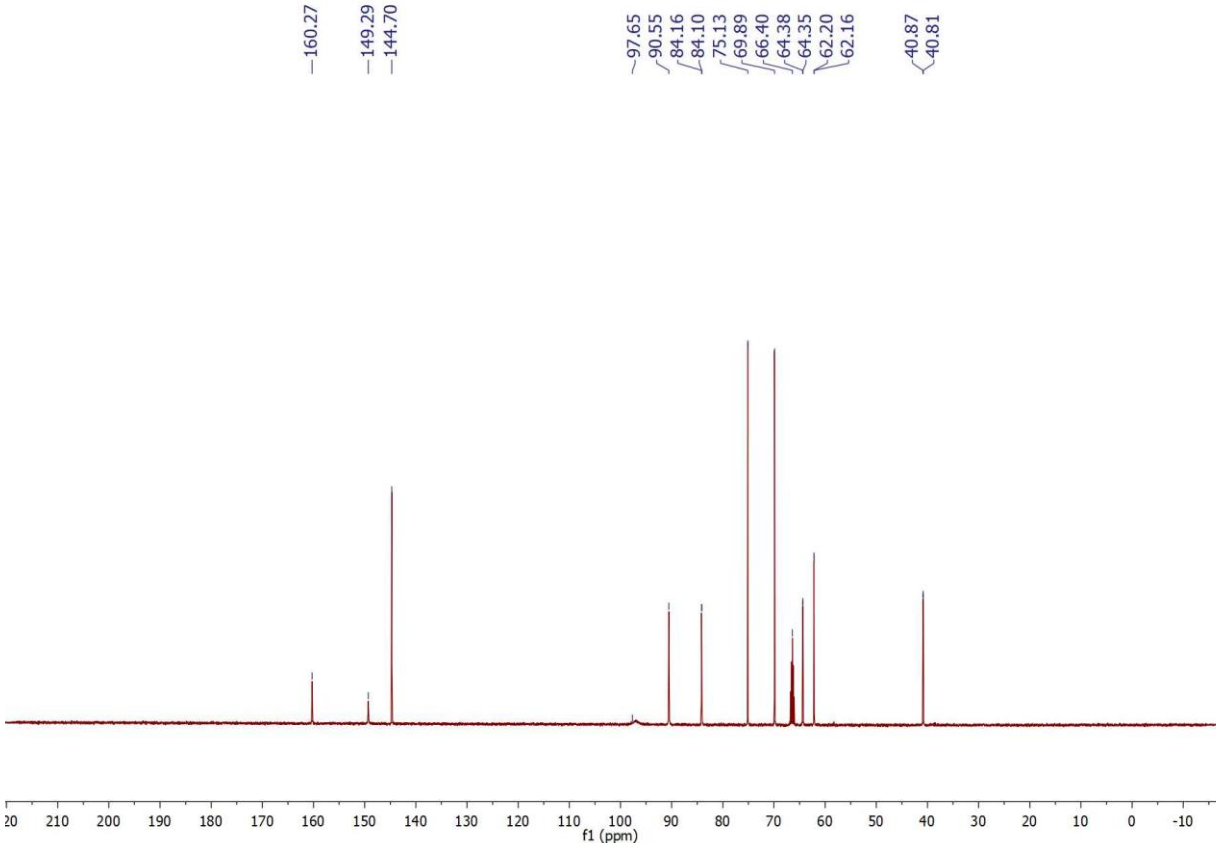

Figure S19. Cu(enP)H_4(CMP) ^{13}C NMR spectrum

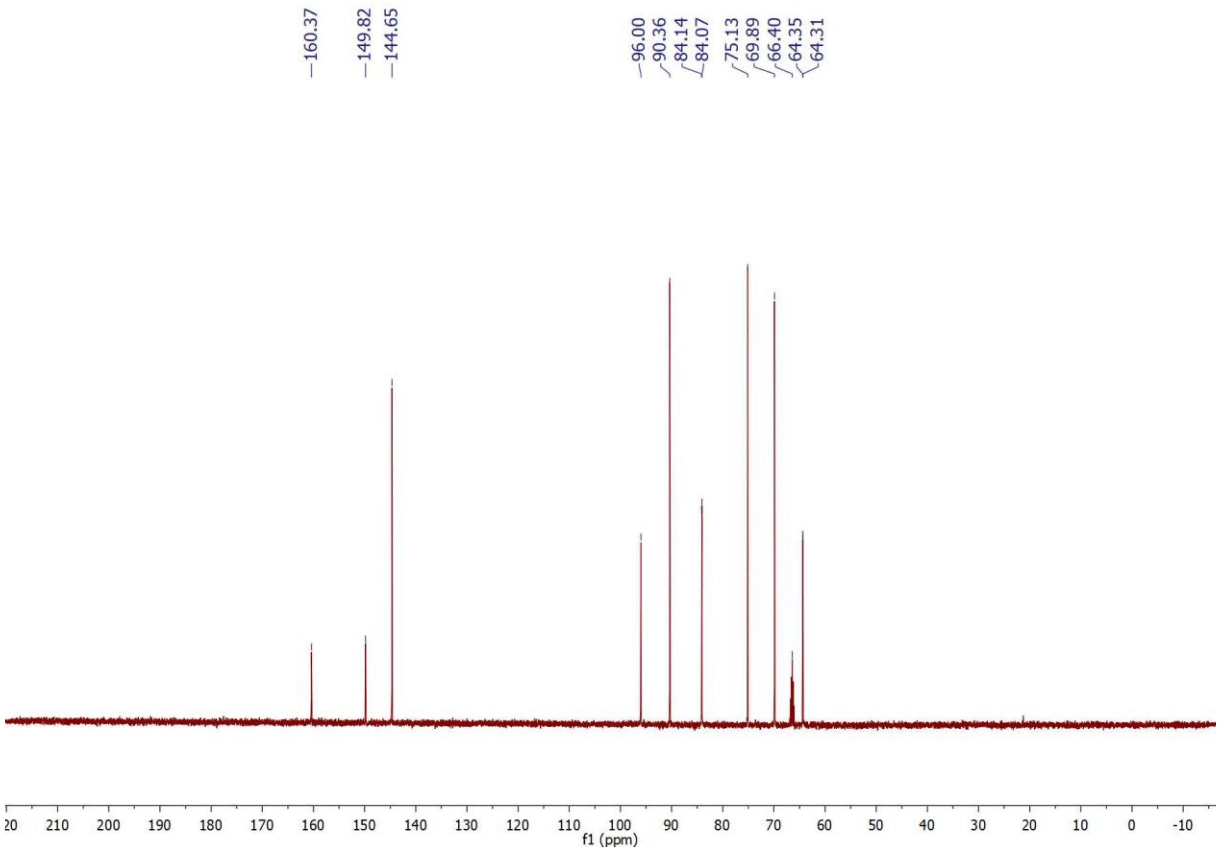

Figure S20. CMP [13] C NMR spectrum

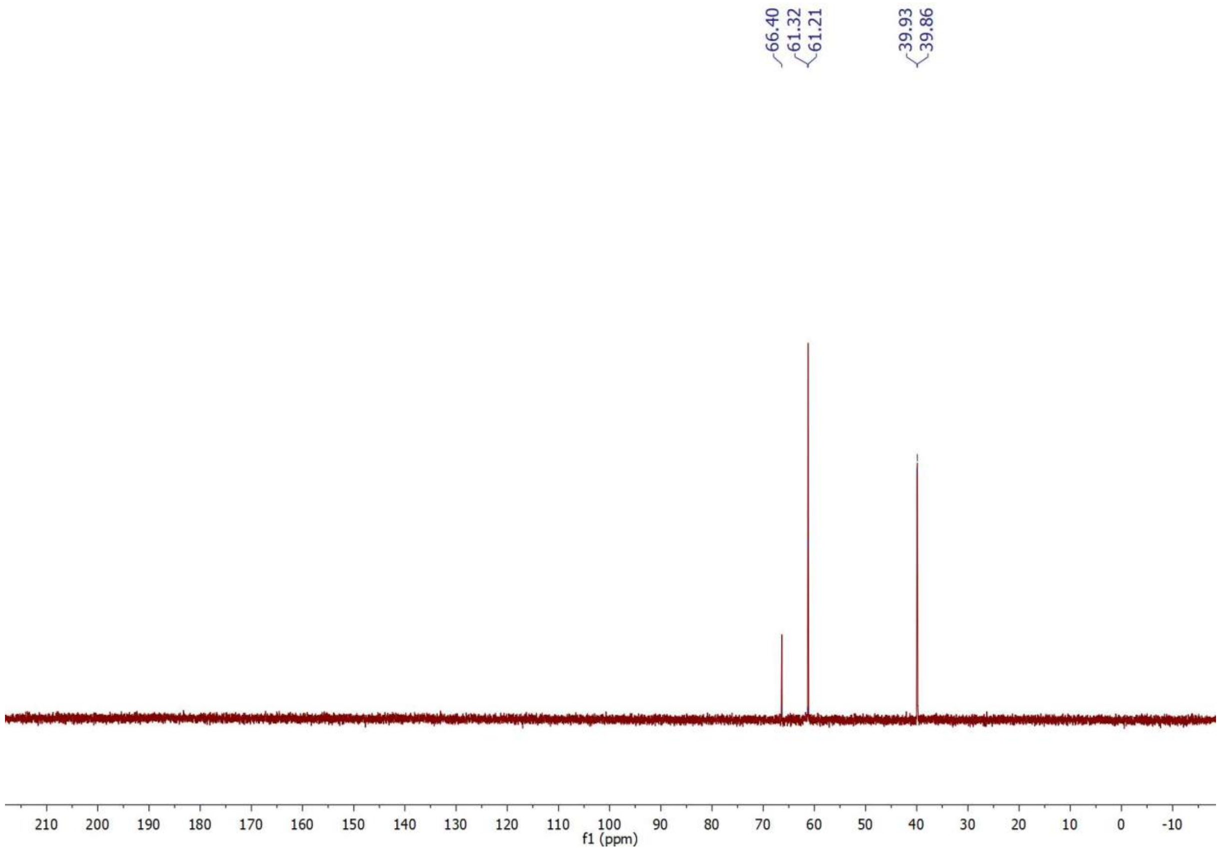

Figure S21. enP [13] C NMR spectrum

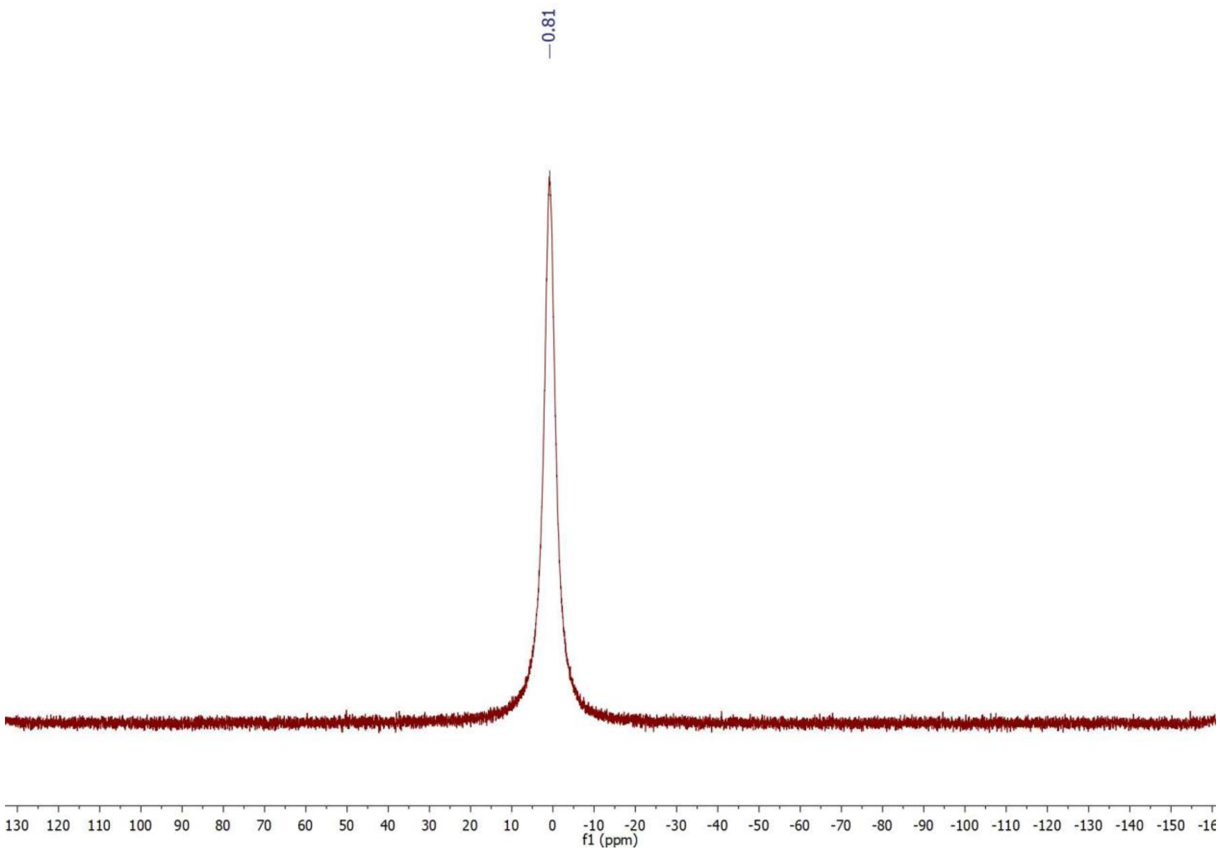

Figure S22. Cu(enP)H_4(CMP) [31] P NMR spectrum

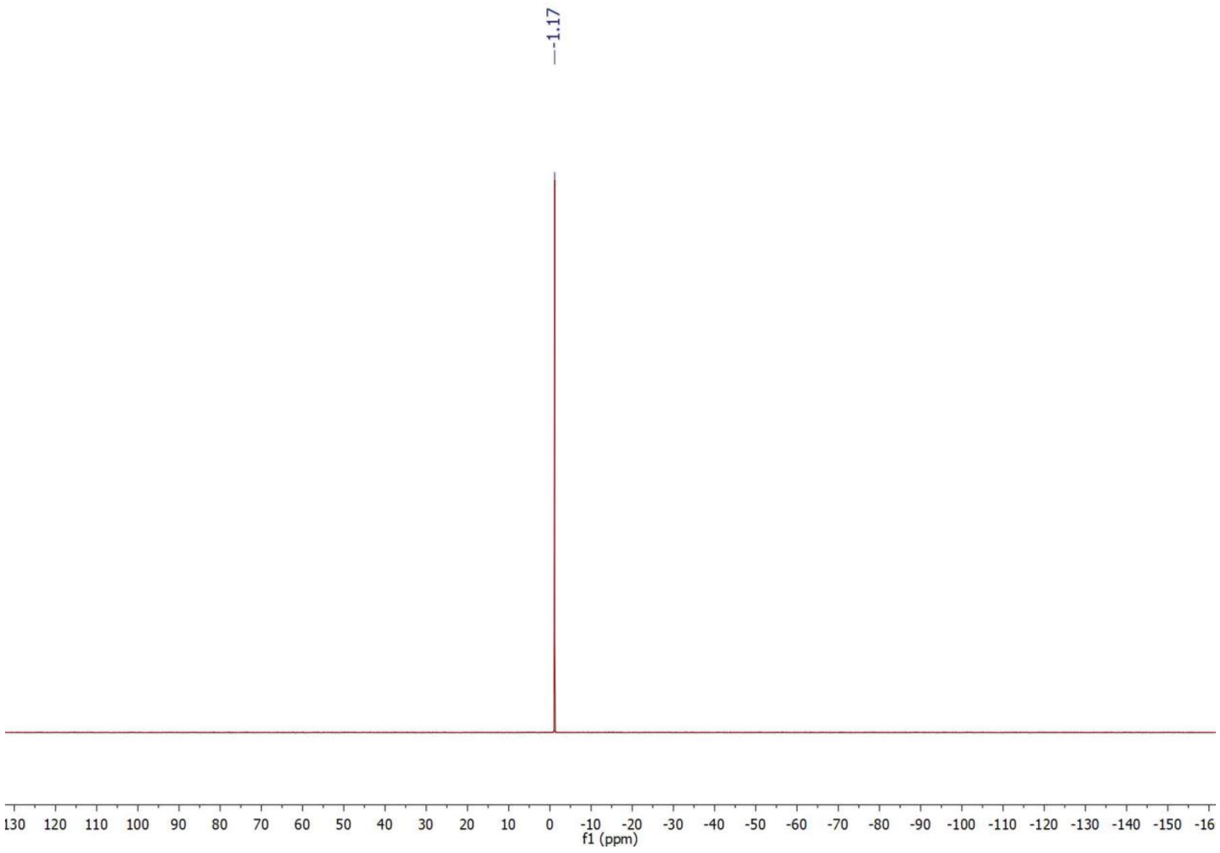

Figure S23. CMP [31] P NMR spectrum

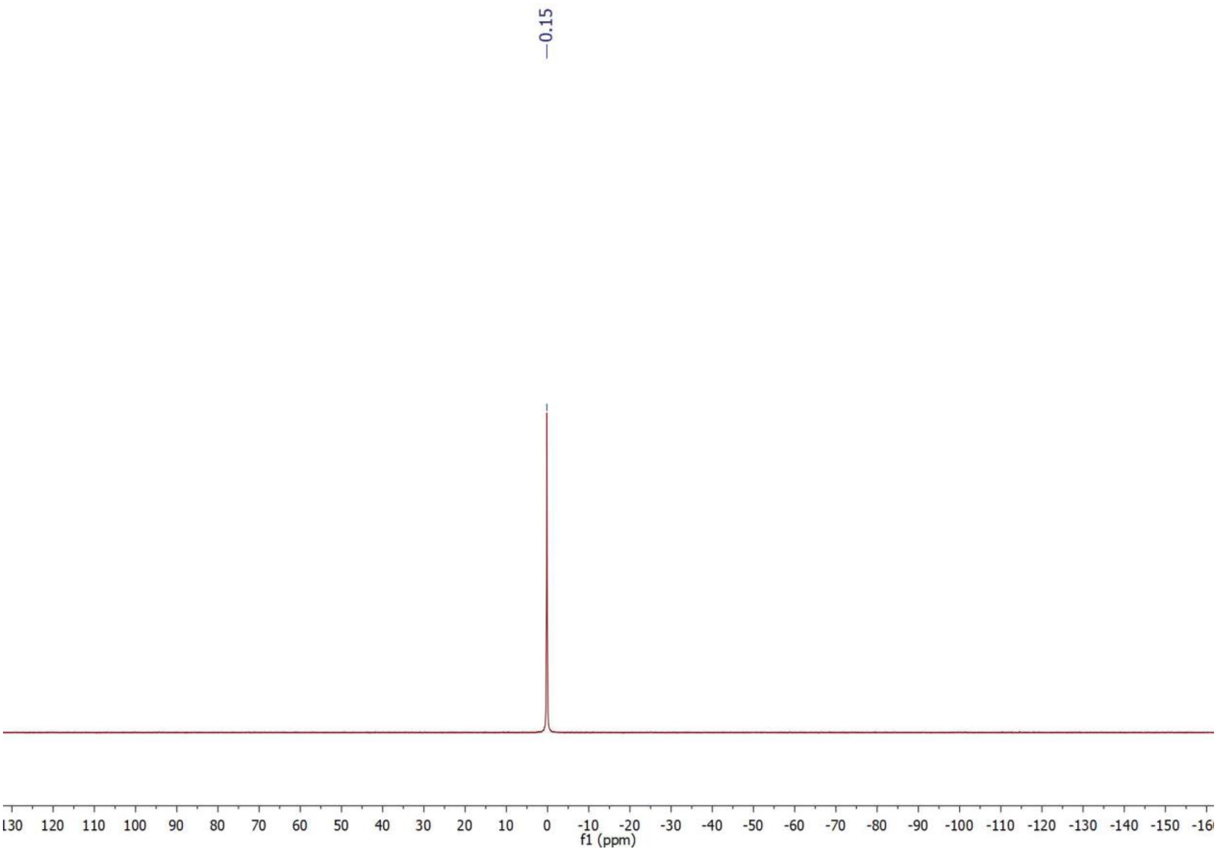

Figure S24. enP [31] NMR spectrum
